# Airway driving pressure and lung stress in ARDS patients

**DOI:** 10.1186/s13054-016-1446-7

**Published:** 2016-08-22

**Authors:** Davide Chiumello, Eleonora Carlesso, Matteo Brioni, Massimo Cressoni

**Affiliations:** 1 Dipartimento di Emergenza-Urgenza, ASST Santi Paolo e Carlo, Milan, Italy; 2 Dipartimento di Scienze della Salute, Università degli Studi di Milano, Milan, Italy; 3 Dipartimento di Fisiopatologia medico-chirurgica e dei Trapianti, Università degli Studi di Milano, Milan, Italy

**Keywords:** ARDS, Lung stress, Driving pressure, VILI, Esophageal pressure, Mortality

## Abstract

**Background:**

Lung-protective ventilation strategy suggests the use of low tidal volume, depending on ideal body weight, and adequate levels of PEEP. However, reducing tidal volume according to ideal body weight does not always prevent overstress and overstrain. On the contrary, titrating mechanical ventilation on airway driving pressure, computed as airway pressure changes from PEEP to end-inspiratory plateau pressure, equivalent to the ratio between the tidal volume and compliance of respiratory system, should better reflect lung injury. However, possible changes in chest wall elastance could affect the reliability of airway driving pressure. The aim of this study was to evaluate if airway driving pressure could accurately predict lung stress (the pressure generated into the lung due to PEEP and tidal volume).

**Methods:**

One hundred and fifty ARDS patients were enrolled. At 5 and 15 cmH_2_O of PEEP, lung stress, driving pressure, lung and chest wall elastance were measured.

**Results:**

The applied tidal volume (mL/kg of ideal body weight) was not related to lung gas volume (*r*^2^ = 0.0005 *p* = 0.772). Patients were divided according to an airway driving pressure lower and equal/higher than 15 cmH_2_O (the lower and higher airway driving pressure groups). At both PEEP levels, the higher airway driving pressure group had a significantly higher lung stress, respiratory system and lung elastance compared to the lower airway driving pressure group. Airway driving pressure was significantly related to lung stress (*r*^2^ = 0.581 *p* < 0.0001 and *r*^2^ = 0.353 *p* < 0.0001 at 5 and 15 cmH_2_O of PEEP). For a lung stress of 24 and 26 cmH_2_O, the optimal cutoff value for the airway driving pressure were 15.0 cmH_2_O (ROC AUC 0.85, 95 % CI = 0.782–0.922); and 16.7 (ROC AUC 0.84, 95 % CI = 0.742–0.936).

**Conclusions:**

Airway driving pressure can detect lung overstress with an acceptable accuracy. However, further studies are needed to establish if these limits could be used for ventilator settings.

**Electronic supplementary material:**

The online version of this article (doi:10.1186/s13054-016-1446-7) contains supplementary material, which is available to authorized users.

## Background

Lung-protective ventilation strategy, commonly employed for moderate to severe forms of acute respiratory distress syndrome (ARDS) [1], suggests the use of low tidal volume, set according to the ideal body weight of the patient (6 mL/kg_IBW_) [2], and higher levels of positive end-expiratory pressure (PEEP) to limit ventilator-induced lung injury (VILI) [2–7]. From a physical point of view, this strategy should minimize the mechanical end-inspiratory lung stress (the applied force), strain (the magnitude of lung deformation) and the opening and closing trauma [8, 9]. It has been reported that VILI develops proportionally to the external energy applied by the ventilator to the lung, mainly due to the dynamic strain and stress caused by tidal volume [10–14]. However, reducing the tidal volume on the basis of ideal body weight, according to the current recommendations, does not always prevent VILI [15–17]. In addition, the selection of optimal PEEP level is still questionable [7, 18–23].

In ARDS, due to the presence of lung disease, the lung available for ventilation is significantly and not uniformly reduced among patients [24]; consequently, a similar tidal volume, based on ideal body weight, can generate different lung stress/strain [25]. On the contrary, titrating the mechanical ventilation on the airway driving pressure, measured as the airway pressure changes from PEEP to end-inspiratory plateau pressure, equivalent to the ratio between the tidal volume and compliance of respiratory system, should better reflect the lung injury because in each patient the applied tidal volume is related to the available lung gas volume [11, 24]. Recently, Amato et al. found that, in ARDS patients ventilated with different combinations of tidal volume and PEEP levels, the airway driving pressure was the factor most strongly related to the outcome [3]. Thus, the airway driving pressure could be a useful tool to identify patients at risk of VILI. In addition, the estimation of airway driving pressure is simpler than that of lung stress, because it does not require the measurement of esophageal pressure by a dedicated balloon, which, for several reasons, is not routinely clinically performed [26]. However, due to the presence of possible alterations in chest wall and lung elastance, the same inspiratory airway pressure can be generated by different tidal volumes [25]. In the presence of an increase in chest wall elastance, the same tidal volume can generate different transpulmonary pressure. Consequently, the airway driving pressure could not adequately reflect lung stress (dynamic plus static stress). On the contrary, the transpulmonary driving pressure (dynamic stress), taking into account the chest wall elastance, could better reflect lung stress.

The aim of this study was to evaluate whether airway driving pressure accurately predicted the dynamic or the static component of lung stress during a PEEP trial with a constant low tidal volume in sedated and paralyzed ARDS patients.

## Methods

### Study population

A total of 150 ARDS patients were included: 21 were enrolled from a new prospective study evaluating the relationship between opening pressure (recruitment pressure) and closing pressure (PEEP) by lung computed tomography (CT) scan (http://clinicaltrials.gov/show/NCT01670747), while 129 were previously enrolled in three published studies [22, 25, 27]. All the studies were approved by the institutional review board of the hospital and written consent was obtained according to current regulations.

#### Study design

All patients were deeply sedated, paralyzed and ventilated in volume-control mode with a tidal volume of 6–8 mL per kilogram of ideal body weight throughout the study protocol. The oxygen fraction, tidal volume and respiratory rate were maintained unchanged for the entire study. Five and 15 cmH_2_O of PEEP were randomly tested. Immediately before the changes of PEEP, a recruitment manoeuvre was performed to standardize the lung volume history. The recruitment manoeuvre was performed in pressure control ventilation at PEEP 5 cmH_2_O, with a plateau pressure of 45 cmH_2_O, inspiratory to expiratory time ratio (I:E) 1:1, and a respiratory rate of 10 breaths/minute for 2 minutes [28]. At each PEEP level, after 20 minutes, respiratory mechanics and blood gas analyses were measured.

### Measurements

#### Respiratory mechanics

The respiratory flow rate was measured with a heated pneumotachograph (Fleisch n°2, Fleisch, Lausanne, Switzerland). Airway pressure (Paw) was measured after the Y piece proximally to the endotracheal tube with a dedicated pressure transducer (MPX 2010 DP, Motorola, Solna, Sweden). Esophageal pressure (Pes) was measured with a radio-opaque balloon (SmartCath, Bicore, Irvine, CA, USA), inflated with 1.0–1.5 mL of air, connected to a pressure transducer. To ensure the correct position of the catheter, the esophageal balloon was positioned in the stomach to check for the presence of positive deflection. Then, it was retracted until it reached the lower third of the esophagus between a depth of 35–40 cm; in this position, an inspiratory occlusion was made to check for concordant changes in airway and esophageal pressure [29]. The balloon inflation was periodically checked to ensure it contained the recommended amount of air. All traces were sampled at 100 Hz and processed on a dedicated data acquisition system (Colligo and Computo, www.elekton.it).

At each PEEP level, the static airway and esophageal pressure were measured during an inspiratory and expiratory pause. Subsequently, disconnecting the ventilator and allowing the respiratory system to deflate from PEEP down to atmospheric pressure (i.e. at functional residual capacity), esophageal pressure was measured. At 5 cmH_2_O in 58 patients the lung gas volume was measured by a simplified helium dilution technique as previously described [30]. The other 92 patients were transported to the radiological department for a lung CT scan, and end-expiratory lung volume at PEEP 5 cmH_2_O was computed by quantitative analysis (see below).

#### Driving pressure, lung stress and partitioned elastance

Airway driving pressure was calculated according to Amato et al. [3], as the airway pressure changes from PEEP to end-inspiratory plateau pressure. It is equivalent to the ratio between the tidal volume and compliance of respiratory system.

The transpulmonary driving pressure, lung stress, respiratory system, lung and chest wall elastance were computed according to the following formula [25, 31]:

$$ \mathrm{Transpulmonary}\ \mathrm{driving}\ \mathrm{pressure}\ \left({\mathrm{cmH}}_2\mathrm{O}\right)=\left[\mathrm{Airway}\ \mathrm{pressure}\ \mathrm{plateau}\kern0.75em \left({\mathrm{cmH}}_2\mathrm{O}\right)-\mathrm{Airway}\ \mathrm{pressure}\ \mathrm{PEEP}\kern0.5em \left({\mathrm{cmH}}_2\mathrm{O}\right)\right] - \left[\mathrm{Esophageal}\ \mathrm{pressure}\ \mathrm{plateau}\kern0.75em \left({\mathrm{cmH}}_2\mathrm{O}\right)-\mathrm{Esophageal}\ \mathrm{pressure}\ \mathrm{PEEP}\kern0.5em \left({\mathrm{cmH}}_2\mathrm{O}\right)\right] $$

$$ \mathrm{Lung}\ \mathrm{stress}\ \left({\mathrm{cmH}}_2\mathrm{O}\right) = \left[\mathrm{Airway}\ \mathrm{pressure}\ \mathrm{plateau}\ \left({\mathrm{cmH}}_2\mathrm{O}\right)-\mathrm{Atmospheric}\ \mathrm{pressure}\kern0.5em \left({\mathrm{cmH}}_2\mathrm{O}\right)\right] - \left[\mathrm{Esophageal}\ \mathrm{pressure}\ \mathrm{plateau}\kern0.75em \left({\mathrm{cmH}}_2\mathrm{O}\right)-\mathrm{Esophageal}\ \mathrm{pressure}\ \mathrm{atmospheric}\ \mathrm{pressure}\kern0.5em \left({\mathrm{cmH}}_2\mathrm{O}\right)\right] $$

$$ \mathrm{Respiratory}\ \mathrm{s}\mathrm{ystem}\ \mathrm{elastance}\ \left(\mathrm{E}\mathrm{r}\mathrm{s}\right)\ \left(\raisebox{1ex}{${\mathrm{cmH}}_2\mathrm{O}$}\!\left/ \!\raisebox{-1ex}{$\mathrm{L}$}\right.\right)=\frac{\mathrm{Airway}\ \mathrm{pressure}\ \mathrm{plateau}\ \left({\mathrm{cmH}}_2\mathrm{O}\right)-\mathrm{Airway}\ \mathrm{pressure}\ \mathrm{PEEP}\left({\mathrm{cmH}}_2\mathrm{O}\right)}{\mathrm{Tidal}\ \mathrm{volume}\ \left(\mathrm{L}\right)} $$

$$ \mathrm{Lung}\ \mathrm{elastance}\ \left(\mathrm{El}\right)\ \left(\raisebox{1ex}{${\mathrm{cmH}}_2\mathrm{O}$}\!\left/ \!\raisebox{-1ex}{$\mathrm{L}$}\right.\right)=\frac{\mathrm{Transpulmonary}\ \mathrm{driving}\ \mathrm{pressure}\ \left({\mathrm{cmH}}_2\mathrm{O}\right)}{\mathrm{Tidal}\ \mathrm{volume}\ \left(\mathrm{L}\right)} $$

$$ \mathrm{Chest}\ \mathrm{w}\mathrm{all}\ \mathrm{elastance}\ \left(\mathrm{E}\mathrm{c}\mathrm{w}\right)\ \left(\raisebox{1ex}{${\mathrm{cmH}}_2\mathrm{O}$}\!\left/ \!\raisebox{-1ex}{$\mathrm{L}$}\right.\right)=\frac{\mathrm{Esophageal}\ \mathrm{pressure}\ \mathrm{plateau}\ \left({\mathrm{cmH}}_2\mathrm{O}\right)-\mathrm{Esophageal}\ \mathrm{pressure}\ \mathrm{PEEP}\left({\mathrm{cmH}}_2\mathrm{O}\right)}{\mathrm{Tidal}\ \mathrm{volume}\ \left(\mathrm{L}\right)} $$

#### Lung CT scan and quantitative analysis

In 91 patients two whole-lung CT scans were performed after a recruitment manoeuvre. During an end-expiratory pause at 5 cmH_2_O of PEEP and an end-inspiratory pause at 45 cmH_2_O of airway pressure, lung CT scans were taken using the following parameters: 110 mAs, tube voltage 120 kV, rotation time 0.5 s, collimation 128 × 0.6 mm, pitch 0.85, and reconstruction matrix 512 × 512. An automatic tube current modulation technique (Care Dose 4, Siemens Medical Solutions, Malvern, PA, USA) allowing for a dynamic reduction of dose radiation during CT examination was applied. In each of the CT slices, lung profiles were manually delineated and analysed using a dedicated software package (Soft-E-Film, www.softefilm.eu). The total lung gas volume, weight, the amount in the different compartments (not inflated, poorly inflated, well inflated and overinflated) and lung recruitability were computed as previously described [28].

#### Statistical analysis

Data are presented as median and interquartile range. The whole population was divided in two groups according to the airway driving pressure lower and equal/higher than 15 cmH_2_O [3]. Physiological variables were compared with unpaired *t* test or Mann-Whitney rank sum test according to the result of the Shapiro-Wilk normality test. Categorical variables were compared with the chi-square test. The role of baseline variables (gas exchange and lung mechanics) on patients outcome from the intensive care unit was assessed with logistic regression analysis (odds ratio [OR] and 95 % confidence intervals [CI]). The agreement between results was assessed using linear regression. A receiver operating characteristics (ROC) curve was used to assess airway and transpulmonary driving pressure ability to predict a lung stress greater than 24 or 26 cmH_2_O [32]. Statistical analysis was performed with SigmaPlot 12.0 (Systat Software, San Jose, CA, USA), logistic regression with SAS statistical software 9.2 (SAS Institute, Cary, NC, USA).

## Results

The main characteristics of the 150 enrolled patients are shown in Table 1. Sixty-five (43 %), 75 (50 %) and ten (7 %) patients presented mild, moderate and severe ARDS. Forty-eight patients (32 %) died in intensive care. At intensive care unit (ICU) admission, non-survivor patients had a higher arterial carbon dioxide (43.1 [36.0–48.3] vs 38.1 [35.0–43.6] mmHg, *p* = 0.019), respiratory rate (16.5 [14.0–20.0] vs 13.0 [11.0–16.5] breaths/min, *p* < 0.01), airway driving pressure at 5 cmH_2_O of PEEP (13.8 [10.7–16.4] vs 12.2 [10.2–14.3] cmH_2_O, *p* = 0.047) and lower PaO_2_/FiO_2_ (160 [117–170] vs 211 [156–257], *p* < 0.0001) compared to survivors (Additional file 1: Table S1). According to multivariate logistic regression, baseline PaO_2_/FiO_2_ (OR 0.989, CI 0.983–0.996; *p* = 0.0015) and baseline respiratory rate (OR 1.090, CI 1.008–1.180; *p* = 0.0315) predicted outcome from ICU, whereas baseline PaCO_2_ (*p* = 0.214) and driving pressures (*p* = 0.453) did not.

**Table 1:** Baseline characteristics of the study population

Characteristics	Overall population (*N* = 150)
Age (years)	62 [47–74]
Male sex, N (%)	102 (68.0)
Body mass index (kg/m^2^)	24.7 [22.8–27.7]
ICU mortality, N (%)	48 (32.0)
Cause of lung injury, N (%):	
• Sepsis	46 (30.7)
• Pneumonia	56 (37.3)
• Trauma	15 (10.0)
• Aspiration	7 (4.7)
• Other	26 (17.3)
ARDS category at clinical PEEP, N (%)	
• Mild	65 (43.3)
• Moderate	75 (50.0)
• Severe	10 (6.7)
PaO_2_/FiO_2_ ratio	187 [146–230]
PaCO_2_ (mmHg)^a^	39.3 [35.1–45.3]
Respiratory rate (bpm)^b^	14 [12–18]
Minute ventilation (L/min)^b^	8.0 [6.6–9.7]
Tidal volume (mL/kg_IBW_)^b^	8.1 [6.7–9.3]
PEEP (cmH_2_O)^a^	10 [10–13]

*ARDS* acute respiratory distress syndrome, *PEEP* positive end-expiratory pressure, *PaO*_*2*_ arterial partial pressure of oxygen, *FiO*_*2*_ inspired fraction of oxygen, *PaCO*_*2*_arterial partial pressure of carbon dioxide, *IBW* ideal body weight

^a^Data available in 143 patients

^b^Data available in 142 patients

### End expiratory lung gas volume

**Fig. 1:**
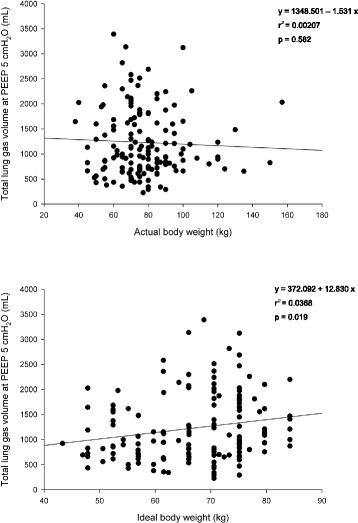
Linear regression between lung gas volume at PEEP 5 cmH_2_O (determined at end-expiration with either lung CT scan or helium dilution technique) and actual body weight (*upper panel*) and ideal body weight (*lower panel*). *PEEP* positive end-expiratory pressure

**Fig. 2:**
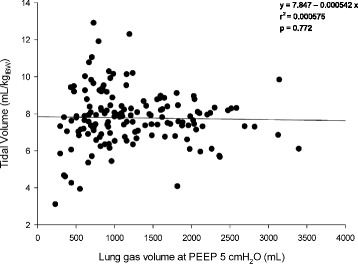
Linear regression between tidal volume (mL/kg of ideal body weight) and lung gas volume at PEEP 5 cmH_2_O (mL). *PEEP* positive end-expiratory pressure

The lung gas volume at PEEP 5 cmH_2_O was 1058 [721–1662] mL and ranged between 229 to 3393 mL. The lung gas volume measured at 5 cmH_2_O of PEEP was not related to the actual body weight (*r*^2^ = 0.002 *p* = 0.58, Fig. 1 upper panel) and poorly related to ideal body weight (*r*^2^ = 0.037 *p* = 0.019, Fig. 1 lower panel). The applied tidal volume standardized for the ideal body weight was not related to the lung gas volume (*r*^2^ = 0.001 *p* = 0.772, Fig. 2).

The respiratory system elastance computed at PEEP 5 cmH_2_O was significantly related to the lung gas volume measured at the same level of PEEP (*r*^2^ = 0.234 *p* < 0.0001) (Additional file 1: Figure S1) and to the well-aerated lung tissue (*r*^2^ = 0.267 *p* < 0.0001) (Additional file 1: Figure S2).

### Respiratory mechanics, lung stress and driving pressure

The population was classified in two groups according to an airway driving pressure lower and equal/higher of 15 cmH_2_O (the lower and higher driving pressure groups). The two groups were similar for age, body mass index and severity of disease (Additional file 1: Table S2).

At 5 cmH_2_O the higher driving pressure group had a significantly higher transpulmonary driving pressure, lung stress, respiratory system elastance, lung elastance, and mortality, and lower lung gas volume compared to the lower driving pressure group (Table 2). At 15 cmH_2_O of PEEP, the higher driving pressure group presented a significantly higher transpulmonary driving pressure, lung stress, and lung and chest wall elastance (Table 3). Gas exchange was similar between the two groups.

**Table 2:** Respiratory mechanics, gas exchange, CT scan variables and outcome of the patients divided according to lower or higher airway driving pressure at PEEP 5 cmH_2_O

Variable	Overall population (*N* = 150)	Lower airway driving pressure (<15 cmH_2_O) (*N* = 108)	Higher airway driving pressure (≥15 cmH_2_O) (*N* = 42)	*p* value
Airway driving pressure (cmH_2_O)	12.6 [10.3–15.2]	11.6 [10.0–13.1]	16.9 [15.7–18.9]	<0.001
Transpulmonary driving pressure (cmH_2_O)	9.0 [7.3–11.7]	8.1 [6.5–9.8]	13.6 [11.7–15.1]	<0.001^a^
End-inspiratory airway plateau pressure (cmH_2_O)	18.2 [16.2–20.9]	17.2 [15.6–18.7]	22.5 [20.9–24.0]	<0.001
Lung stress (cmH_2_O)	13.5 [10.7–16.0]	11.8 [10.0–14.0]	17.5 [15.4–18.9]	<0.001^a^
Respiratory system elastance (cmH_2_O/L)	25.2 [19.8–30.5]	22.1 [18.7–26.7]	33.6 [29.6–40.4]	<0.001
Lung elastance (cmH_2_O/L)	17.5 [13.9–23.2]	15.4 [12.7–19.9]	27.4 [21.9–31.6]	<0.001
Chest wall elastance (cmH_2_O/L)	6.3 [4.3–9.0]	5.9 [4.3–8.7]	7.8 [4.6–10.4]	0.054
PaCO_2_ (mmHg)^b^	44.2 [39.9–50.5]	43.9 [39.2–50.5]	47.6 [41.3–50.7]	0.117^a^
PaO_2_/FiO_2_ ratio^b^	143 [98–177]	145 [104–178]	132 [81–176]	0.404
Lung total gas (mL)^c^	1058 [721–1662]	1234 [879–1827]	694 [562–903]	<0.001
Total lung tissue weight (g)^b^	1394 [1145–1684]	1369 [1173–1742]	1457 [1050–1682]	0.787
• Non-aerated lung tissue (%)^b^	45.2 [34.0–56.9]	44.9 [36.6–53.6]	50.4 [30.7–61.1]	0.419^a^
• Poorly aerated lung tissue (%)^b^	28.1 [20.2–39.2]	27.1 [20.1–36.1]	34.1 [20.3–43.0]	0.067
• Well-aerated lung tissue (%)^b^	23.9 [14.2–33.9]	28.2 [17.2–34.6]	16.5 [8.2–26.7]	0.006
• Over-aerated lung tissue (%)^b^	0.01 [0.00–0.17]	0.03 [0.00–0.29]	0.00 [0.00–0.02]	0.002
Lung recruitability (%)^b^	15.6 [7.9–23.7]	12.5 [7.5–22.1]	18.5 [9.7–26.4]	0.110
ICU mortality N (%)	48 (32.0)	29 (26.9)	19 (45.2)	0.049

Lung mechanics, gas exchange, CT-related variables and lung total gas were determined at PEEP 5 cmH_2_O. Statistical analysis: Student’s *t* test^a^, Mann-Whitney rank sum test, chi-square, as appropriate

*Abbreviations*: *CT* computed tomography, *PEEP* positive end-expiratory pressure, *PaCO*_*2*_ arterial partial pressure of carbon dioxide, *PaO*_*2*_ arterial partial pressure of oxygen, *FiO*_*2*_ inspired fraction of oxygen, *ICU* intensive care unit

^b^Data available for 91 patients (59 in the “lower airway driving pressure” group and 32 in the “higher airway driving pressure” group)

^c^Total gas was computed either by CT scan analysis (in 59 and 32 patients, respectively), or by helium dilution technique (in 48 and ten patients, respectively)

**Table 3:** Respiratory mechanics, gas exchange and outcome of the patients divided according to lower or higher airway driving pressure at PEEP 15 cmH_2_O

Variable	Overall population (*N* = 150)	Lower airway driving pressure (<15 cmH_2_O) (*N* = 97)	Higher airway driving pressure (≥15 cmH_2_O) (*N* = 53)	*p* value
Airway driving pressure (cmH_2_O)	13.2 [11.2–16.9]	11.9 [10.2–13.0]	18.0 [16.6–19.9]	<0.001
Transpulmonary driving pressure (cmH_2_O)	9.5 [7.8–12.2]	8.4 [6.6–9.7]	13.1 [10.4–15.5]	<0.001
End-inspiratory airway plateau pressure (cmH_2_O)	28.4 [25.8–31.3]	26.7 [24.8–28.2]	32.9 [30.8–35.1]	<0.001
Lung stress (cmH_2_O)	20.7 [17.9–23.1]	19.3 [16.8–21.7]	23.9 [20.8–26.2]	<0.001
Respiratory system elastance (cmH_2_O/L)	26.3 [21.2–32.2]	22.8 [19.5–26.6]	34.2 [29.6–41.9]	<0.001
Lung elastance (cmH_2_O/L)	18.7 [14.5–24.2]	16.7 [13.1–19.7]	24.6 [19.5–31.3]	<0.001
Chest wall elastance (cmH_2_O/L)	7.0 [4.8–10.3]	6.2 [4.6–8.2]	10.2 [5.9–12.3]	<0.001
PaCO_2_ (mmHg)^a^	45 [40.2–50.4]	43.8 [39.0–49]	46.8 [41.9–53.2]	0.039
PaO_2_/FiO_2_ ratio^a^	178 [137–237]	175 [136–230]	187 [146–257]	0.314
ICU mortality N (%)	48 (32.0)	28 (28.9)	20 (37.7)	0.352

Lung mechanics and gas exchange variables were determined at PEEP 15 cmH_2_O. Statistical analysis: Mann-Whitney rank sum test, chi-square, as appropriate

*Abbreviations*: *PEEP* positive end-expiratory pressure, *PaCO*_*2*_ arterial partial pressure of carbon dioxide, *PaO*_*2*_ arterial partial pressure of oxygen, *FiO*_*2*_ inspired fraction of oxygen, *ICU* intensive care unit

^a^Data available in 91 patients (64 in the “lower airway driving pressure” group and 27 in the “higher airway driving pressure” group)

The patients (*N* = 23, 21.3 %) who, increasing PEEP to 15 cmH_2_O, were reassigned from the lower driving pressure group to the higher driving pressure group, had (at PEEP 5 cmH_2_O) a higher airway driving pressure, transpulmonary driving pressure and lung stress compared patients remaining in the lower driving pressure group (Additional file 1: Table S3).

### Driving pressure and lung stress

**Fig. 3:**
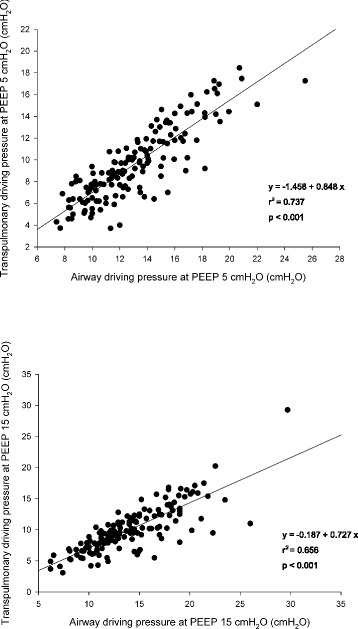
Linear regression between transpulmonary and airway driving pressure (cmH_2_O) at PEEP 5 (*upper panel*) and 15 cmH_2_O (*lower panel*). *PEEP* positive end-expiratory pressure

**Fig. 4:**
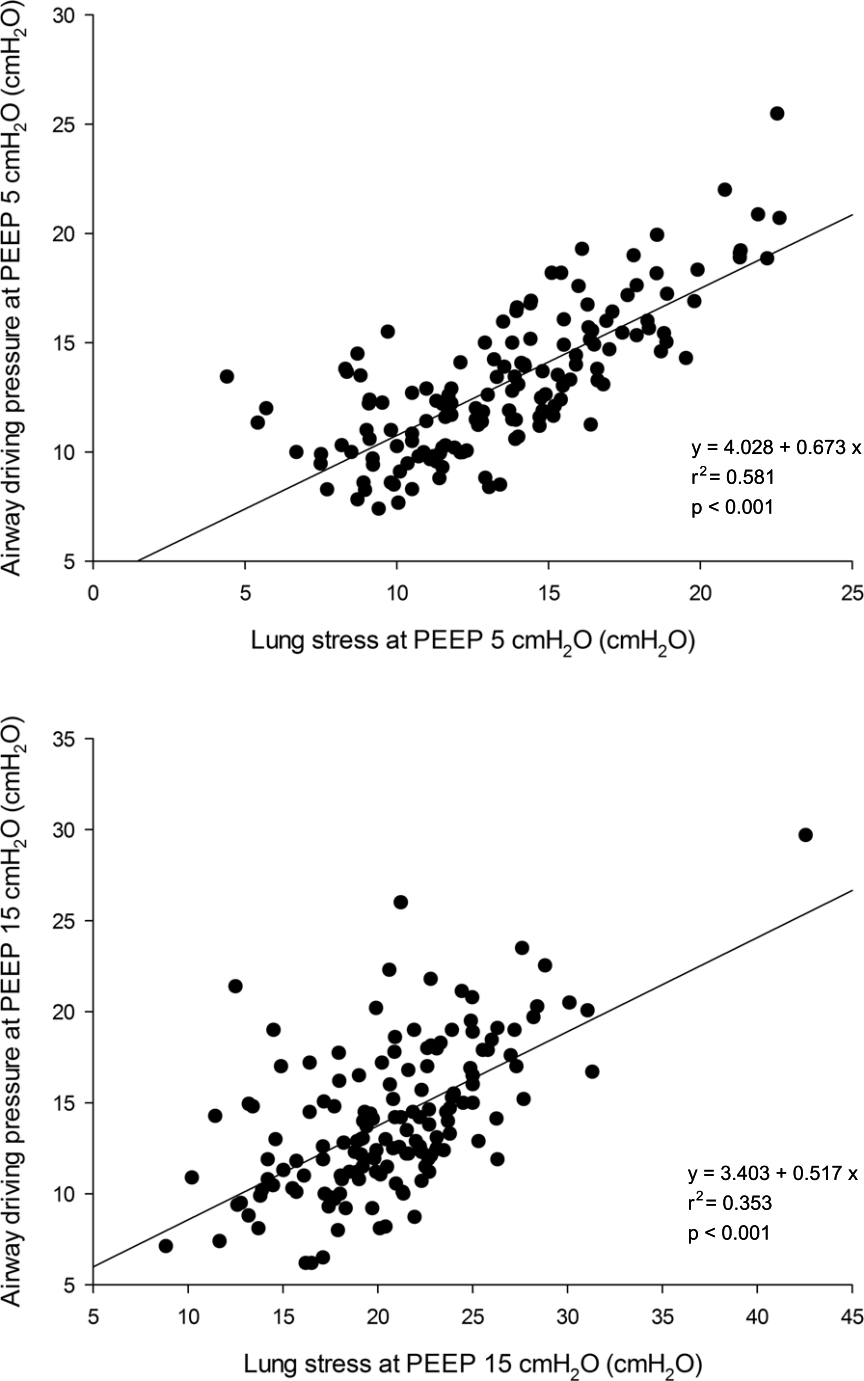
Linear regression between airway driving pressure (cmH_2_O) and lung stress (cmH_2_O) at PEEP 5 (*upper panel*) and 15 cmH_2_O (*lower panel*). *PEEP* positive end-expiratory pressure

**Fig. 5:**
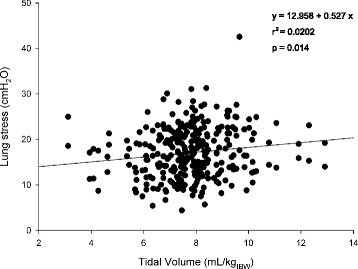
Linear regression between lung stress (cmH_2_O) and the applied tidal volume (mL/kg of ideal body weight)

The transpulmonary driving pressure was significantly related to the airway driving pressure (*r*^2^ = 0.737 *p* < 0.0001, and *r*^2^ = 0.656 *p* < 0.0001 at 5 and 15 cmH_2_O of PEEP, respectively; Fig. 3). The airway driving pressure was significantly related to lung stress (*r*^2^ = 0.581 *p* < 0.0001 and *r*^2^ = 0.353 *p* < 0.0001 at 5 and 15 cmH_2_O of PEEP, respectively; Fig. 4). Similarly, also the transpulmonary driving pressure was significantly related to lung stress (*r*^2^ = 0.854 *p* < 0.0001 and *r*^2^ = 0.668 *p* < 0.0001 at 5 and 15 cmH_2_O of PEEP, respectively) (Additional file 1: Figure S3). The lung stress was not related to the applied tidal volume (*r*^2^ = 0.010 *p* = 0.08, Fig. 5).

Additional linear regression is reported in the Additional file 1: Figures S4, S5.

### ROC analysis

**Fig. 6:**
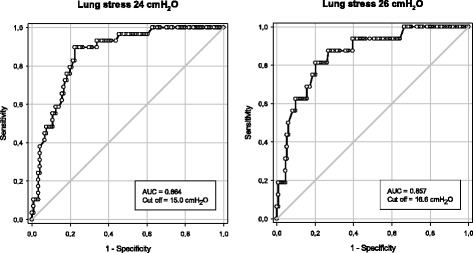
Receiver operator characteristic (ROC) curve for airway driving pressure as a predictor of lung stress above 24 (*left panel*) or 26 cmH_2_O (*right panel*). *AUC* area under the curve

At 5 cmH_2_O of PEEP no patient had a lung stress above 24 cmH_2_O; therefore, ROC analysis was performed only considering airway and transpulmonary driving pressures at PEEP 15 cmH_2_O. Optimal cutoff values for airway driving pressure were 15.0 cmH_2_O (area under the curve [AUC] = 0.864, 95 %CI 0.801–0.929) and 16.6 cmH_2_O (AUC = 0.864, 95 %CI 0.767–0.947), considering a stress equal or above 24 and 26 cmH_2_O, respectively (Fig. 6). For transpulmonary driving pressure, optimal cutoff values were 11.7 cmH_2_O (AUC = 0.962, 95 %CI 0.934–0.989) and 11.8 cmH_2_O (AUC = 0.938, 95 %CI 0.894–0.982), considering a stress equal or above 24 and 26 cmH_2_O, respectively (Additional file 1: Figure S6, Table S4).

## Discussion

The primary findings of this study, aimed to assess the relationship between driving pressure and lung stress, are that: (1) at both the tested levels of PEEP (5 and 15 cmH_2_O) patients with a higher driving pressure presented a significantly higher lung stress; (2) the airway driving pressure was sufficiently accurate to detect lung stress higher than 24 and 26 cmH_2_O.

ARDS is commonly managed by invasive mechanical ventilation. Unfortunately, the mechanical ventilation can further damage the lung, activating a biological inflammatory response and promoting VILI [8, 11, 33]. In order to limit VILI, ensuring at the same time adequate ventilation, a lung-protective ventilation strategy has been implemented [2–7]. Presently, there are no clear thresholds for tidal volume or plateau pressure that may ensure a safe ventilator strategy [34, 35], and these recommendations are not widely applied [36]. Trying to reduce overstress/strain, the majority of the studies applied a tidal volume standardized to ideal body weight, computed according to the patient’s height and sex [37], as in healthy subjects the lung volume is related to height [38]. Thus, an obese patient should not receive a higher tidal volume just because of the weight gain, compared to a normal body weight patient with similar height. Unlike healthy subjects, in ARDS patients the amount of lung gas volume in which the tidal volume is distributed is highly variable, and depends on the severity of the disease [25, 28]. In the present study, the tidal volumes set according both to the ideal body weight and to the actual body weight were not related to the lung gas volume. Consequently, a similar amount of tidal volume could produce different stress among different patients with similar body weight (Fig. 5). One possible solution should be to titrate the tidal volume according to the airway driving pressure, which depends on the respiratory system elastance, thus better reflecting the severity of the disease [3]. To estimate the “true” driving pressure, it is necessary to have the patients well relaxed, with or without paralysis, to avoid any possible respiratory effort. Amato et al., retrospectively analysing the airway driving pressure from the individual data of 3562 ARDS deeply sedated patients, enrolled in more than nine clinical trials, showed that driving pressure was strongly associated with outcome [3]. Similarly, Cinnella et al, applying an open lung approach based on a recruitment manoeuvre followed by a decremental PEEP trial, compared to the ARDS Network protocol, significantly reduced the driving pressure with a higher tidal volume fraction inflating the dorsal lung regions [39]. These studies showed the possibility of using airway driving pressure as a possible surrogate of lung stress at bedside [39, 40] avoiding thus the use of esophageal manometry and any disconnection from the ventilator that can be technically challenging [26].

However, due to the possible impairment of chest wall elastance in ARDS [41, 42], a similar airway pressure can be associated to a significantly different transpulmonary pressure, depending on the relationship between lung and chest wall elastance [25, 43]. In an experimental animal study with and without abdominal hypertension at different PEEP levels, the airway driving pressure paralleled the behaviours of transpulmonary driving pressure; however, their ratio was always lower than 1, with a mean range of 0.45 to 0.79 [31].

In the present study, we divided the population according to an airway driving pressure lower than, equal or higher than 15 cmH_2_O, a value that was associated to an increase in the mortality in the study published by Amato et al. [3]. At both PEEP levels, the lower driving pressure group had a significantly lower stress; furthermore, patients who were reclassified from lower to higher airway driving pressure group by passing from 5 to 15 cmH_2_O, presented higher lung stress and lung elastance. Regarding the possibility that airway driving pressure could discriminate patients with lung overstress, although several studies titrated adjusted mechanical ventilation on the basis of lung stress, there is not a clear threshold at the present time; thus, in this study, two different cutoffs, 24 and 26 cmH_2_O of transpulmonary pressure, were considered [21, 25, 42, 44, 45]. However, experimental studies both in sheep [46, 47] and in piglets [13, 48, 49] show that VILI occurs if mechanical ventilation is conducted in the range of total lung capacity. Ratio of inspiratory capacity to resting lung volume (total lung capacity/functional residual capacity, TLC/FRC) is approximately 2.2–2.6 and is highly conserved among species. In normal humans FRC is 2200 mL and TLC 6000 mL [50]; it follows that the physical limit of lung expansion is between 2.5 and 3. We found [25] that specific elastance (defined as the transpulmonary pressure/stress needed to double the FRC) in ARDS and healthy subjects is approximately 13.5 cmH_2_O. It follows that applying a stress of 27 cmH_2_O implies adding a volume of two times the FRC, reaching the total lung capacity. For this reason we proposed two thresholds immediately below the strain of 27 (24 and 26 cmH_2_O). At 5 cmH_2_O of PEEP none of the patients had a lung stress higher than 24 cmH_2_O. This suggests that applying a low tidal volume with low PEEP avoids an unsafe lung stress. However, this approach could be equally harmful in moderate to severe ARDS, where usually lung recruitability is high and cyclic intra-tidal opening and closing would occur [51, 52]. On the contrary, at 15 cmH_2_O of PEEP, 29 patients presented a lung stress higher than 24 and 16 patients higher than 26 cmH_2_O respectively. In the linear regression there was a relative amount of variability suggesting that the exact lung stress could not be predicted with confidence. However, as shown by the ROC curves, the airway driving pressure presented an acceptable sensitivity and specificity to detect lung stress higher than 24 and 26 cmH_2_O (Additional file 1: Table S4).

Contrary to the Amato et al. study, we found a difference in outcome between patients with lower or higher driving pressure only at 5 cmH_2_O of PEEP, but this can be easily explained by the fact that the patients after the study underwent different setting of mechanical ventilation and were not managed according to the driving pressure.

Although respiratory rate, minute ventilation, and inspiratory flow [14, 53] can be associated to lung injury, the total alveolar deformation during mechanical ventilation due to the application of PEEP (static strain) and to the tidal volume (dynamic strain) is the most frequently used indicator of VILI at bedside. Experimental studies showed that dynamic strain is more harmful compared to an equivalent static strain, and furthermore maintaining the same global deformation by a simultaneous reduction of the dynamic component and an increase of the static component caused less VILI [54–57]. Although the monitoring of airway driving pressure is able to detect the possible presence of lung overstress, it does not give any information about the other possible associated factors in modulating VILI [14, 57].

### Limitations

Possible limitations of this study are: (1) the included patients originated by mixing from previous published data [22, 25, 27] and a new prospective study; (2) the majority of the patients presented a mild to moderate form of ARDS.

## Conclusions

Mechanical ventilation, applying pressure and volume with non-physiologic distortion (i.e. strain) and stress (i.e. transpulmonary pressure), can generate an energy load to lung parenchyma, which promotes VILI. The airway driving pressure can be a plausible, non-invasive method to predict lung stress in mild and moderate ARDS. However, further prospective studies are needed to establish the limits of overstress that should be used at bedside.

## Key messages

- The tidal volume based on ideal body weight is not related to the amount of aerated lung volume and to lung stress
- At 5 and 15 cmH_2_O of PEEP patients with a higher airway driving pressure presented a significantly higher lung stress (dynamic plus static stress).
- The airway driving pressure was sufficiently accurate to detect lung stress higher than 24 and 26 cmH_2_O.
- Further prospective studies are needed to establish the limits of overstress.

## Abbreviations

ARDS, acute respiratory distress syndrome; AUC, area under the curve; CI, confidence interval; CT, computed tomography; Ecw, elastance of the chest wall; El, elastance of the lung; Ers, elastance of the respiratory system; FRC, functional residual capacity; IBW, ideal body weight; OR, odds ratio; PEEP, positive end-expiratory pressure; ROC, receiver operator characteristic; TLC, total lung capacity; VILI, ventilation-induced lung injury

## Additional file

Additional file 1: Expanded results with additional tables and figures. (PDF 2454 kb)

## References

[1] Ferguson ND, Fan E, Camporota L, Antonelli M, Anzueto A, Beale R (2012). The Berlin definition of ARDS: an expanded rationale, justification, and supplementary material. Intensive Care Med.

[2] Ventilation with lower tidal volumes as compared with traditional tidal volumes for acute lung injury and the acute respiratory distress syndrome. The Acute Respiratory Distress Syndrome Network. N Engl J Med. 2000;342:1301–8. 10.1056/NEJM200005043421801 10793162

[3] Amato MBP, Meade MO, Slutsky AS, Brochard L, Costa ELV, Schoenfeld DA (2015). Driving pressure and survival in the acute respiratory distress syndrome. N Engl J Med.

[4] Ranieri VM, Rubenfeld GD, Thompson BT, Ferguson ND, Caldwell E, Fan E (2012). Acute respiratory distress syndrome: the Berlin Definition. JAMA.

[5] Villar J, Kacmarek RM, Perez-Mendez L, Aguirre-Jaime A (2006). A high positive end-expiratory pressure, low tidal volume ventilatory strategy improves outcome in persistent acute respiratory distress syndrome: a randomized, controlled trial. Crit Care Med.

[6] Dellinger RP, Levy MM, Rhodes A, Annane D, Gerlach H, Opal SM (2013). Surviving Sepsis Campaign: international guidelines for management of severe sepsis and septic shock, 2012. Intensive Care Med.

[7] Briel M, Meade M, Mercat A, Brower RG, Talmor D, Walter SD (2010). Higher vs lower positive end-expiratory pressure in patients with acute lung injury and acute respiratory distress syndrome: systematic review and meta-analysis. JAMA.

[8] Slutsky AS, Ranieri VM (2013). Ventilator-induced lung injury. N Engl J Med.

[9] Wilson T, American Physiological Society (1986). Solid mechanics. Handbook of physiology: a critical, comprehensive presentation of physiological knowledge and concepts.

[10] Kolobow T (2004). Volutrauma, barotrauma, and ventilator-induced lung injury: lessons learned from the animal research laboratory. Crit Care Med.

[11] Gattinoni L, Carlesso E, Cadringher P, Valenza F, Vagginelli F, Chiumello D (2003). Physical and biological triggers of ventilator-induced lung injury and its prevention. Eur Respir J Suppl.

[12] Gonzalez-Lopez A, Garcia-Prieto E, Batalla-Solis E, Amado-Rodriguez L, Avello N, Blanch L (2012). Lung strain and biological response in mechanically ventilated patients. Intensive Care Med.

[13] Protti A, Cressoni M, Santini A, Langer T, Mietto C, Febres D (2011). Lung stress and strain during mechanical ventilation: any safe threshold?. Am J Respir Crit Care Med.

[14] Cressoni M, Gotti M, Chiurazzi C, Massari D, Algieri I, Amini M (2016). Mechanical power and development of ventilator-induced lung injury. Anesthesiology.

[15] Terragni PP, Rosboch G, Tealdi A, Corno E, Menaldo E, Davini O (2007). Tidal hyperinflation during low tidal volume ventilation in acute respiratory distress syndrome. Am J Respir Crit Care Med.

[16] Gattinoni L, Chiumello D, Carlesso E, Valenza F (2004). Bench-to-bedside review: chest wall elastance in acute lung injury/acute respiratory distress syndrome patients. Crit Care Lond Engl.

[17] Cortes-Puentes GA, Cortes-Puentes LA, Adams AB, Anderson CP, Marini JJ, Dries DJ (2013). Experimental intra-abdominal hypertension influences airway pressure limits for lung protective mechanical ventilation. J Trauma Acute Care Surg.

[18] Meade MO, Cook DJ, Guyatt GH, Slutsky AS, Arabi YM, Cooper DJ (2008). Ventilation strategy using low tidal volumes, recruitment maneuvers, and high positive end-expiratory pressure for acute lung injury and acute respiratory distress syndrome: a randomized controlled trial. JAMA.

[19] Mercat A, Richard J-CM, Vielle B, Jaber S, Osman D, Diehl J-L (2008). Positive end-expiratory pressure setting in adults with acute lung injury and acute respiratory distress syndrome: a randomized controlled trial. JAMA.

[20] Brower RG, Lanken PN, MacIntyre N, Matthay MA, Morris A, Ancukiewicz M (2004). Higher versus lower positive end-expiratory pressures in patients with the acute respiratory distress syndrome. N Engl J Med.

[21] Talmor D, Sarge T, Malhotra A, O’Donnell CR, Ritz R, Lisbon A (2008). Mechanical ventilation guided by esophageal pressure in acute lung injury. N Engl J Med.

[22] Chiumello D, Cressoni M, Carlesso E, Caspani ML, Marino A, Gallazzi E (2014). Bedside selection of positive end-expiratory pressure in mild, moderate, and severe acute respiratory distress syndrome. Crit Care Med.

[23] Guerin C (2011). The preventive role of higher PEEP in treating severely hypoxemic ARDS. Minerva Anestesiol.

[24] Gattinoni L, Pesenti A (2005). The concept of “baby lung”. Intensive Care Med.

[25] Chiumello D, Carlesso E, Cadringher P, Caironi P, Valenza F, Polli F (2008). Lung stress and strain during mechanical ventilation for acute respiratory distress syndrome. Am J Respir Crit Care Med.

[26] Akoumianaki E, Maggiore SM, Valenza F, Bellani G, Jubran A, Loring SH (2014). The application of esophageal pressure measurement in patients with respiratory failure. Am J Respir Crit Care Med.

[27] Chiumello D, Marino A, Cressoni M, Mietto C, Berto V, Gallazzi E (2013). Pleural effusion in patients with acute lung injury: a CT scan study. Crit Care Med.

[28] Gattinoni L, Caironi P, Cressoni M, Chiumello D, Ranieri VM, Quintel M (2006). Lung recruitment in patients with the acute respiratory distress syndrome. N Engl J Med.

[29] Chiumello D, Chidini G, Calderini E, Colombo A, Crimella F, Brioni M (2016). Respiratory mechanics and lung stress/strain in children with acute respiratory distress syndrome. Ann Intensive Care.

[30] Chiumello D, Cressoni M, Chierichetti M, Tallarini F, Botticelli M, Berto V (2008). Nitrogen washout/washin, helium dilution and computed tomography in the assessment of end expiratory lung volume. Crit Care.

[31] Cortes-Puentes GA, Keenan JC, Adams AB, Parker ED, Dries DJ, Marini JJ (2015). Impact of chest wall modifications and lung injury on the correspondence between airway and transpulmonary driving pressures. Crit Care Med.

[32] Hanley JA, McNeil BJ (1982). The meaning and use of the area under a receiver operating characteristic (ROC) curve. Radiology.

[33] Dreyfuss D, Saumon G (1998). Ventilator-induced lung injury: lessons from experimental studies. Am J Respir Crit Care Med.

[34] Deans KJ, Minneci PC, Cui X, Banks SM, Natanson C, Eichacker PQ (2005). Mechanical ventilation in ARDS: one size does not fit all. Crit Care Med.

[35] Hager DN, Krishnan JA, Hayden DL, Brower RG (2005). Tidal volume reduction in patients with acute lung injury when plateau pressures are not high. Am J Respir Crit Care Med.

[36] Jaswal DS, Leung JM, Sun J, Cui X, Li Y, Kern S (2014). Tidal volume and plateau pressure use for acute lung injury from 2000 to present: a systematic literature review. Crit Care Med.

[37] Girard TD, Bernard GR (2007). Mechanical ventilation in ARDS: a state-of-the-art review. Chest.

[38] Ibanez J, Raurich JM (1982). Normal values of functional residual capacity in the sitting and supine positions. Intensive Care Med.

[39] Cinnella G, Grasso S, Raimondo P, D’Antini D, Mirabella L, Rauseo M (2015). Physiological effects of the open lung approach in patients with early, mild, diffuse acute respiratory distress syndrome: an electrical impedance tomography study. Anesthesiology.

[40] Loring SH, Malhotra A (2015). Driving pressure and respiratory mechanics in ARDS. N Engl J Med.

[41] Gattinoni L, Pesenti A, Avalli L, Rossi F, Bombino M (1987). Pressure-volume curve of total respiratory system in acute respiratory failure. Computed tomographic scan study. Am Rev Respir Dis.

[42] Ranieri VM, Brienza N, Santostasi S, Puntillo F, Mascia L, Vitale N (1997). Impairment of lung and chest wall mechanics in patients with acute respiratory distress syndrome: role of abdominal distension. Am J Respir Crit Care Med.

[43] Gattinoni L, Pelosi P, Suter PM, Pedoto A, Vercesi P, Lissoni A (1998). Acute respiratory distress syndrome caused by pulmonary and extrapulmonary disease. Different syndromes?. Am J Respir Crit Care Med.

[44] Grasso S, Terragni P, Birocco A, Urbino R, Del Sorbo L, Filippini C (2012). ECMO criteria for influenza A (H1N1)-associated ARDS: role of transpulmonary pressure. Intensive Care Med.

[45] Grasso S, Mascia L, Del Turco M, Malacarne P, Giunta F, Brochard L (2002). Effects of recruiting maneuvers in patients with acute respiratory distress syndrome ventilated with protective ventilatory strategy. Anesthesiology.

[46] Mandava S, Kolobow T, Vitale G, Foti G, Aprigliano M, Jones M (2003). Lethal systemic capillary leak syndrome associated with severe ventilator-induced lung injury: an experimental study. Crit Care Med.

[47] Kolobow T, Moretti MP, Fumagalli R, Mascheroni D, Prato P, Chen V (1987). Severe impairment in lung function induced by high peak airway pressure during mechanical ventilation. An experimental study. Am Rev Respir Dis.

[48] Cressoni M, Chiurazzi C, Gotti M, Amini M, Brioni M, Algieri I (2015). Lung inhomogeneities and time course of ventilator-induced mechanical injuries. Anesthesiology.

[49] Protti A, Andreis DT, Milesi M, Iapichino GE, Monti M, Comini B (2015). Lung anatomy, energy load, and ventilator-induced lung injury. Intensive Care Med Exp.

[50] American Physiological Society (1986). Handbook of physiology: a critical, comprehensive presentation of physiological knowledge and concepts.

[51] Caironi P, Carlesso E, Cressoni M, Chiumello D, Moerer O, Chiurazzi C (2015). Lung recruitability is better estimated according to the Berlin definition of acute respiratory distress syndrome at standard 5 cm H2O rather than higher positive end-expiratory pressure: a retrospective cohort study. Crit Care Med.

[52] Caironi P, Cressoni M, Chiumello D, Ranieri M, Quintel M, Russo SG (2010). Lung opening and closing during ventilation of acute respiratory distress syndrome. Am J Respir Crit Care Med.

[53] Protti A, Maraffi T, Milesi M, Votta E, Santini A, Pugni P, et al. Role of strain rate in the pathogenesis of ventilator-induced lung edema. Crit. Care Med. 2016. [Epub ahead of print] 10.1097/CCM.0000000000001718 27054894

[54] Tschumperlin DJ, Oswari J, Margulies AS (2000). Deformation-induced injury of alveolar epithelial cells. Effect of frequency, duration, and amplitude. Am J Respir Crit Care Med.

[55] Webb HH, Tierney DF (1974). Experimental pulmonary edema due to intermittent positive pressure ventilation with high inflation pressures. Protection by positive end-expiratory pressure. Am Rev Respir Dis.

[56] Protti A, Votta E, Gattinoni L (2014). Which is the most important strain in the pathogenesis of ventilator-induced lung injury: dynamic or static?. Curr Opin Crit Care.

[57] Protti A, Andreis DT, Monti M, Santini A, Sparacino CC, Langer T (2013). Lung stress and strain during mechanical ventilation: any difference between statics and dynamics?. Crit Care Med.

