# Supplementary material for: Airway driving pressure and lung stress in ARDS patients
Source: Crit Care. 2016 Aug 22;20:276. doi: 10.1186/s13054-016-1446-7 (PMC4993008; doi:10.1186/s13054-016-1446-7)

Airway driving pressure and lung stress in ARDS patients

Davide Chiumello MD[1,2], Eleonora Carlesso MSc[2], Matteo Brioni MD[2], Massimo Cressoni MD[2]

[1] Dipartimento di Anestesia, Rianimazione ed Emergenza-Urgenza, Fondazione IRCCS Ca' Granda - Ospedale Maggiore Policlinico, Milano, Italy

[2] Dipartimento di Fisiopatologia Medico-Chirurgica e dei Trapianti, Università degli Studi di Milano, Italy

Additional file 1

Table S1. Patients characteristic according to intensive care unit outcome.

	Survivors (N=102)	Non-survivors (N=48)	P value
**Age (years)**	61.0 [45.0 – 74.0]	65.5 [52.5 – 72.5]	0.17
**Male sex, N (%)**	73 (71.6)	29 (60.4)	0.239
**Body mass index (kg/m^2)**	24.7 [22.9 – 27.7]	24.6 [22.6 – 30.1]	0.64
**PaO_2/FiO_2 ratio**	211 [156 – 257]	160 [117 – 170]	< 0.0001
**$PaCO_2$ (mmHg)***	38.1 [35.0 – 43.6]	43.1 [36 – 48.3]	0.019
**Respiratory system elastance (cmH_2O/L)°**	22.9 [20.0 – 28.7]	28.1 [23.2 – 35.2]	0.016
**Tidal volume (mL/kg_{IBW})***	8.3 [7.1 – 9.3]	7.8 [6.7 – 8.6]	0.22
**PEEP (cmH_2O)***	10.0 [10.0 – 12.5]	10.0 [10.0 – 14.0]	0.055
**Respiratory rate (breaths/min)***	13.0 [11.0 – 16.5]	16.5 [14.0 – 20.0]	< 0.01
**Airway driving pressure at PEEP 5 cmH_2O (cmH_2O)**	12.2 [10.2 – 14.3]	13.8 [10.7 – 16.4]	0.047
**Transpulmonary driving pressure at PEEP 5 cmH_2O (cmH_2O)**	8.9 [7.0 – 10.9]	10.0 [7.7 – 13.2]	0.057
**Lung stress at PEEP 5 cmH_2O (cmH_2O)**	12.9 [10.7 – 15.5]	14.0 [11.0 – 16.4]	0.32
**Airway driving pressure at PEEP 15 cmH_2O (cmH_2O)**	13.0 [11.2 – 16.5]	14.1 [11.4 – 17.1]	0.37
**Transpulmonary driving pressure at PEEP 15 cmH_2O (cmH_2O)**	9.3 [7.3 – 12.2]	10.0 [8.2 – 12.0]	0.19
**Lung stress at PEEP 15 cmH_2O (cmH_2O)**	20.4 [18.0 – 23.1]	21.1 [17.8 – 23.6]	0.57
**Total gas at PEEP 5 cmH_2O (mL)#**	1133 [783 – 1662]	933 [662 – 1701]	0.20
**Total tissue at PEEP 5 cmH_2O (g)§**	1391[1199 – 1670]	1395 [1074 – 1686]	0.58
• **Not inflated tissue (%)§**	44.9 [38.4 – 57.3]	47.5 [31.2 – 56.1]	0.80
• **Poorly inflated tissue (%)§**	27.9 [20.1 – 38.3]	29.1 [20.4 – 39.7]	0.51
• **Well inflated tissue (%)§**	25.7 [13.8 – 34.3]	22.6 [14.2 – 32.7]	0.42
• **Over inflated tissue (%)§**	0.01 [0.00 – 0.10]	0.02 [0.00 – 0.24]	0.24
**Lung recruitability (%)§**	12.6 [7.0 – 21.9]	16.3 [8.6 – 25.9]	0.268

Statistical Analysis: Student 's t –test, Mann-Whitney Rank sum Test, Chi-Square, as appropriate.
Abbreviations: PaO_2: arterial partial pressure of oxygen; FiO_2: inspired fraction of oxygen; $PaCO_2$: arterial partial pressure of carbon dioxide; PEEP: positive end-expiratory pressure.
* Data available for 97 patients in the "survivors" group and for 46 patients in the "non-survivors" group.
° Data available for 42 patients in the "survivors" group and for 30 patients in the "non-survivors" group.
# Total gas was computed by CT scan analysis (for 53 in the "survivor" group, and for 38 patients in the "non-survivors" group, respectively), or by helium dilution technique (for 48 in the "survivor" group, and for 10 patients in the "non-survivors" group, respectively).
§ Data available for 53 patients in the "survivors" group and for 38 patients in the "non-survivors" group.

Table S2. Baseline characteristics of the study population divided according to lower or higher airway driving pressure at PEEP 5 cmH_2O.

Characteristics	Overall population (N=150)	Lower airway driving pressure (<15 cmH_2O) (N=108)	Higher airway driving pressure (≥15 cmH_2O) (N=42)	P value
**Age (years)**	62 [47 – 74]	64 [49 – 74]	57 [45 – 75]	0.339
**Male sex, N (%)**	102 (68.0)	75 (69.4)	27 (51.9)	0.679
**Body Mass Index (kg/m^2)**	24.7 [22.8 – 27.7]	24.2 [22.5 – 27.5]	25.9 [23.9 – 29.0]	0.041
**ICU mortality, N (%)**	48 (32.0)	29 (26.9)	19 (45.2)	0.049
**Cause of lung injury, N (%):**				0.739
• **Sepsis**	46 (30.7)	36 (33.3)	10 (23.8)	
• **Penumonia**	56 (37.3)	40 (37.0)	16 (38.1)	
• **Trauma**	15 (10.0)	10 (9.3)	5 (11.9)	
• **Aspiration**	7 (4.7)	4 (3.7)	3 (7.1)	
• **Other**	26 (17.3)	18 (16.7)	8 (19.0)	
**ARDS category at PEEP 5 cmH_2O, N(%):**	(n=91)	(n=59)	(n=32)	0.553
• **Mild**	16 (17.6)	11 (18.6)	5 (15.6)	
• **Moderate**	50 (54.9)	34 (57.6)	16 (50.0)	
• **Severe**	25 (27.5)	14 (23.7)	11 (34.4)	

Statistical Analysis: Mann-Whitney Rank Sum Test, Chi-Square, as appropriate.

Abbreviations: ICU: intensive care unit; ARDS: acute respiratory distress syndrome; PEEP: positive end-expiratory pressure.

Table S3. Respiratory mechanics, gas exchange, CT scan variables and outcome of the patients divided according to lower or higher airway driving pressure at PEEP 5 cmH_2O and 15 cmH_2O.

	Variable	Lower airway driving pressure (<15 cmH_2O) at PEEP 15 cmH_2O	Higher airway driving pressure (≥15 cmH_2O) at PEEP 15 cmH_2O	P value
Lower airway driving pressure (<15 cmH_2O) at PEEP 5 cmH_2O	**Number of patients**	**85**	**23**	
	**Airway driving pressure (cmH_2O)**	11.0 [9.7 – 12.4]	13.5 [12.2 – 14.0]	<0.001
	**Transpulmonary driving pressure (cmH_2O)**	7.8 [6.2 – 9.2]	9.7 [7.7 – 10.7]	0.002§
	**End-Inspiratory airway plateau pressure (cmH_2O)**	16.7 [15.1 – 18.0]	20.0 [18.0 – 21.1]	<0.001
	**Lung stress (cmH_2O)**	11.5 [9.8 – 13.7]	13.8 [11.5 – 15.5]	0.003§
	**Respiratory system elastance (cmH_2O/L)**	21.3 [17.8 – 25.5]	26.5 [21.3 – 27.7]	0.011
	**Lung elastance (cmH_2O/L)**	14.8 [12.6 – 18.0]	19.8 [14.7 – 21.0]	0.028
	**Chest wall elastance (cmH_2O/L)**	5.6 [4.1 – 8.8]	6.2 [4.9 – 8.4]	0.467
	**$PaCO_2$ (mmHg)***	43.9 [39.5 – 50.6]	42.0 [36.9 – 48.2]	0.467
	**PaO_2/FiO_2 ratio***	143 [99 – 170]	212 [136 – 300]	0.004§
	**Total lung gas (mL)°**	1236 [839 – 1912]	1210 [944 – 1669]	0.900
	**Total lung tissue weight (g)***	1397 [1212 – 1832]	1200 [1115 – 1338]	0.077
	• **Not-aerated lung tissue (%)***	45.9 [35.9 – 56.2]	39.4 [37.0 – 42.2]	0.167§
	• **Poorly-aerated lung tissue (%)***	27.1 [20.0 – 37.3]	29.9 [21.7 – 34.0]	0.950
	• **Well-aerated lung tissue (%)***	27.4 [15.9 – 34.4]	31.5 [29.8 – 39.3]	0.066§
	• **Over-aerated lung tissue (%)***	0.03 [0.00 – 0.29]	0.08 [0.01 – 0.11]	0.716
	**Lung recruitability (%)***	12.6 [7.9 – 23.4]	5.6 [2.5 – 18.3]	0.123
	**ICU mortality N (%)**	24 (28.2)	5 (21.7)	0.720
Higher airway driving pressure (≥15 cmH_2O) at PEEP 5 cmH_2O	**Number of patients**	**12**	**30**	
	**Airway driving pressure (cmH_2O)**	15.6 [15.4 – 16.6]	17.4 [16.4 – 19.1]	0.013
	**Transpulmonary driving pressure (cmH_2O)**	12.8 [10.8 – 14.0]	14.2 [11.8 – 15.1]	0.215§
	**End-Inspiratory airway plateau pressure (cmH_2O)**	21.0 [20.3 – 21.8]	23.0 [21.4 – 24.3]	0.010
	**Lung stress (cmH_2O)**	16.9 [15.4 – 18.9]	17.7 [15.4 – 19.8]	0.389§
	**Respiratory system elastance (cmH_2O/L)**	33.4 [28.4 – 38.0]	33.8 [29.8 – 40.4]	0.707
	**Lung elastance (cmH_2O/L)**	27.4 [20.4 – 32.7]	27.1 [21.8 – 31.6]	0.967
	**Chest wall elastance (cmH_2O/L)**	7.0 [4.6 – 9.5]	8.2 [4.5 – 11.6]	0.646
	**$PaCO_2$ (mmHg)#**	41.6 [40.1 – 47.6]	50.0 [43.2 – 53.3]	0.016§
	**PaO_2/FiO_2 ratio#**	113 [80 – 210]	134 [82 – 176]	0.958§
	**Total lung gas (mL)+**	665 [471 – 812]	726 [562 – 960]	0.351
	**Total lung tissue weight (g)#**	1449 [1107 – 1649]	1466 [1046 – 1702]	0.685§
	• **Not-aerated lung tissue (%)#**	52.4 [26.9 – 64.7]	48.7 [33.5 – 58.9]	0.886§
	• **Poorly-aerated lung tissue (%)#**	27.3 [21.1 – 43.6]	38.0 [20.2 – 42.5]	0.466§
	• **Well-aerated lung tissue (%)#**	16.8 [9.3 – 27.2]	15.0 [8.1 – 26.3]	0.704§
	• **Over-aerated lung tissue (%)#**	0.00 [0.00 – 0.01]	0.00 [0.00 – 0.04]	0.905
	**Lung recruitability (%)#**	17.2 [6.1 – 28.6]	19.9 [10.4 – 26.2]	0.550§
	**ICU mortality N (%)**	4 (33.3)	15 (50.0)	0.524

Lung mechanics, gas exchange, CT-related variables and lung total gas were determined at PEEP 5 cmH_2O.

Statistical Analysis: Student's t-test§, Mann-Whitney Rank Sum Test, Chi-Square, as appropriate.

Abbreviations: PaO_2: arterial partial pressure of oxygen; FiO_2: inspired fraction of oxygen; $PaCO_2$: arterial partial pressure of carbon dioxide; ICU: intensive care unit; PEEP: positive end-expiratory pressure.

* Data available for 53 patients in the "lower airway driving pressure at PEEP 15 cmH_2O" group and for 6 patients in the "higher airway driving pressure at PEEP 15 cmH_2O" group.

° Total gas was computed either by CT scan analysis (in 53 patients of the "lower airway driving pressure at PEEP 15 cmH_2O" group, and in 6 patients of the "higher airway driving pressure at PEEP 15 cmH_2O" group, respectively), or by helium dilution technique (in 31 patients of the "lower airway driving pressure at PEEP 15 cmH_2O" group, and in 17 patients of the "higher airway driving pressure at PEEP 15 cmH_2O" group, respectively).

[#] Data available for 11 patients in the "lower airway driving pressure at PEEP 15 cmH_2O" group and for 21 patients in the "higher airway driving pressure at PEEP 15 cmH_2O" group.

[+] Total gas was computed either by CT scan analysis (in 11 patients of the "lower airway driving pressure at PEEP 15 cmH_2O" group, and in 21 patients of the "higher airway driving pressure at PEEP 15 cmH_2O" group, respectively), or by helium dilution technique (in one patient of the "lower airway driving pressure at PEEP 15 cmH_2O" group, and in 9 patients of the "higher airway driving pressure at PEEP 15 cmH_2O" group, respectively).

Table S4. Sensibility, specificity, positive and negative predictive values of optimal cut off for airway and transpulmonary driving pressure to predict a lung stress equal or above 24 or 26 cmH_2O.

	**Sensitivity**	**Specificity**	**Positive predictive value**	**Negative predictive value**
Lung stress equal or above 24 cmH_2O				
**Airway driving pressure (cut off 15.0 cmH_2O)**	0.90	0.78	0.80	0.88
**Transpulmonary driving pressure (cut off 11.7 cmH_2O)**	0.97	0.89	0.90	0.96
Lung stress equal or above 26 cmH_2O				
**Airway driving pressure (cut off 16.6 cmH_2O)**	0.81	0.80	0.80	0.81
**Transpulmonary driving pressure (cut off 11.8 cmH_2O)**	0.94	0.82	0.84	0.93

Figure S1. Linear regression between respiratory system elastance (cmH_2O/L) and lung gas volume at PEEP 5 cmH_2O (mL).

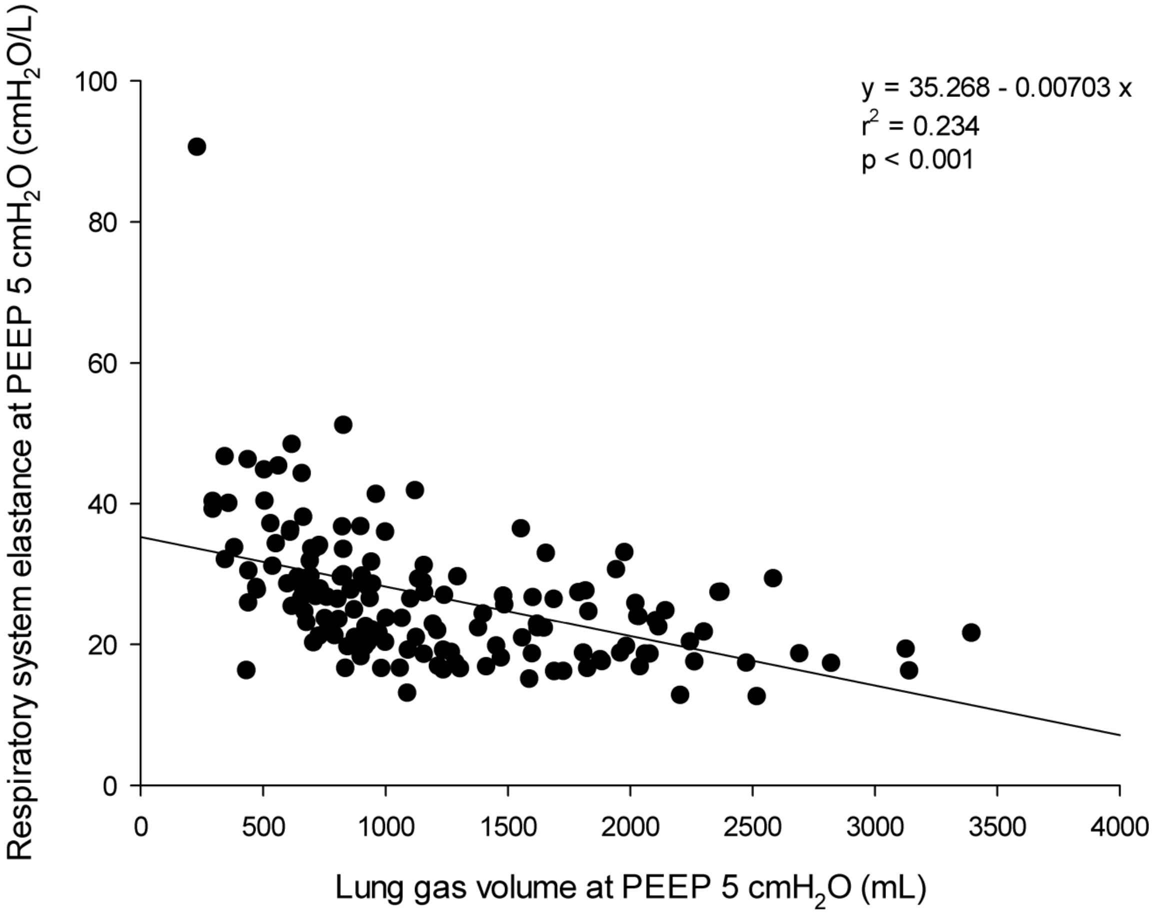

Figure S2. Linear regression between respiratory system elastance (cmH_2O/L) and well-aerated lung tissue weight at PEEP 5 cmH_2O (g).

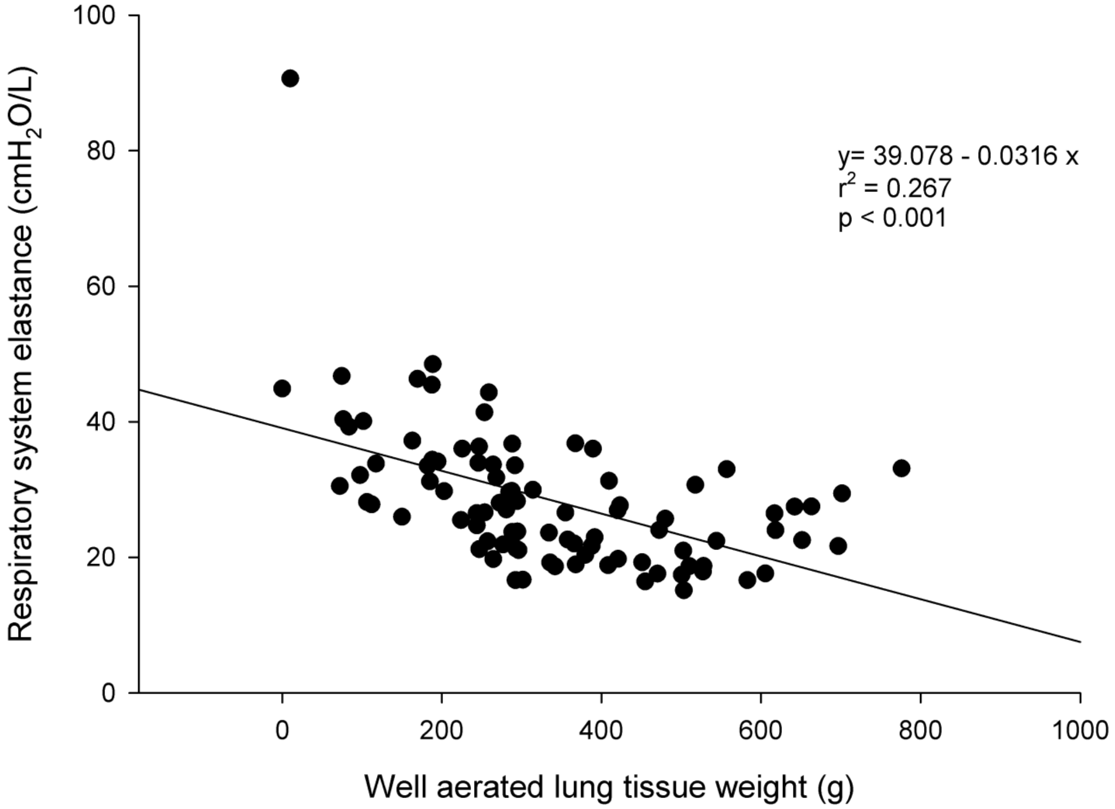

Figure S3. Linear regression between transpulmonary driving pressure (cmH$_2$O) and lung stress (cmH$_2$O) at PEEP 5 (upper panel) and 15 cmH$_2$O (lower panel).

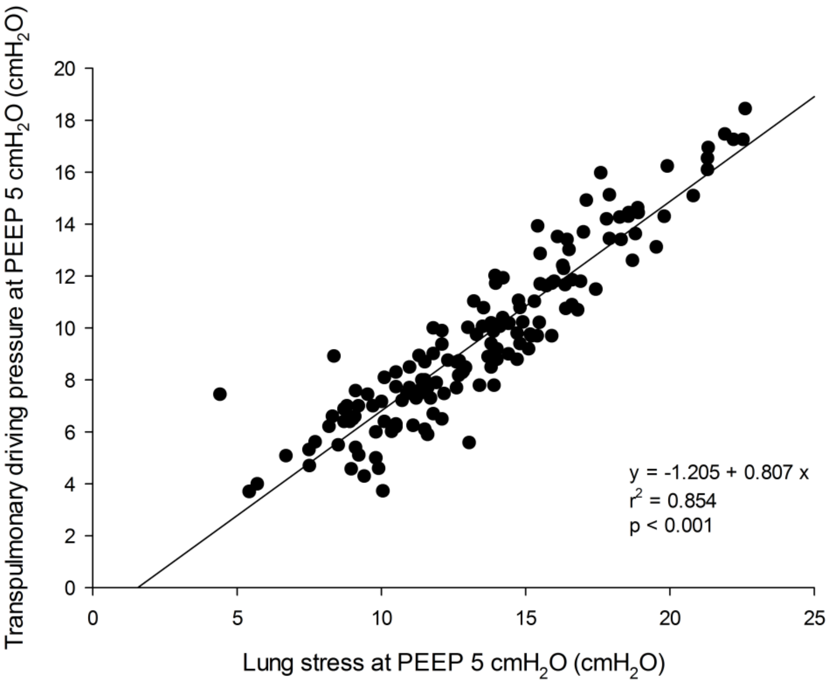

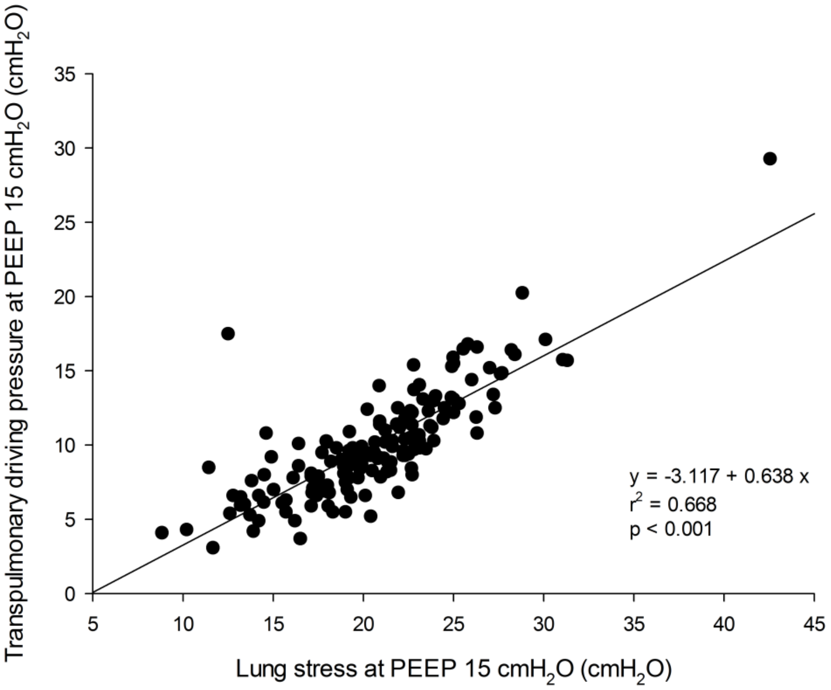

Figure S4. Linear regression between lung stress (cmH_2O) and airway end-inspiratory plateau pressure (cmH_2O).

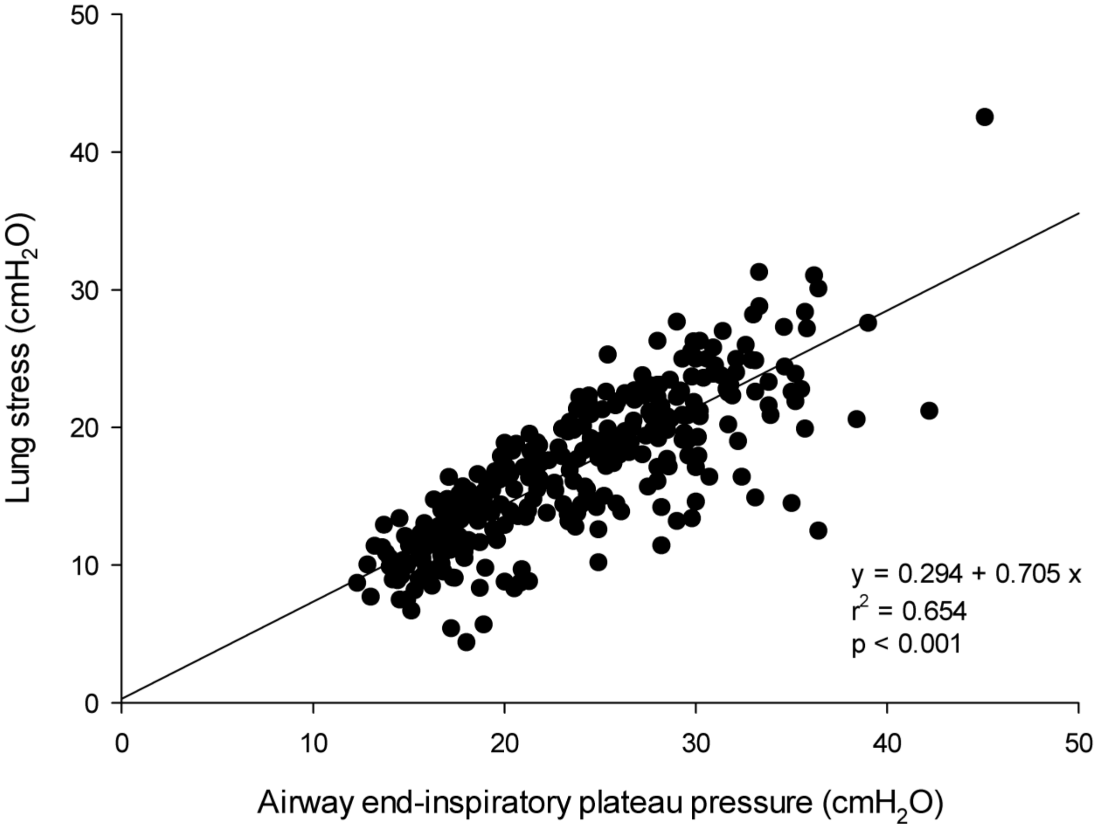

Figure S5. Linear regression between airway driving pressure (cmH_2O) and lung gas volume (mL) at PEEP 5 cmH_2O.

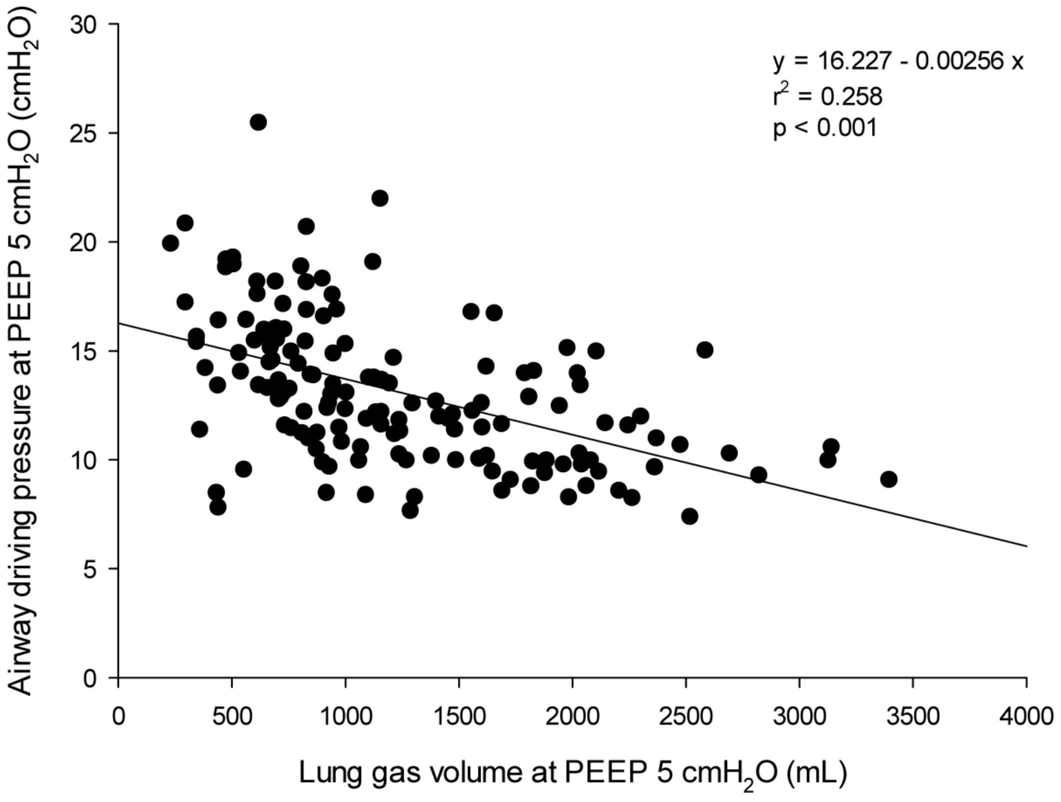

Figure S6. Receiver operator characteristic (ROC) curve for transpulmonary driving pressure as a predictor of lung stress above 24 (left panel) or 26 cmH_2O (right panel).

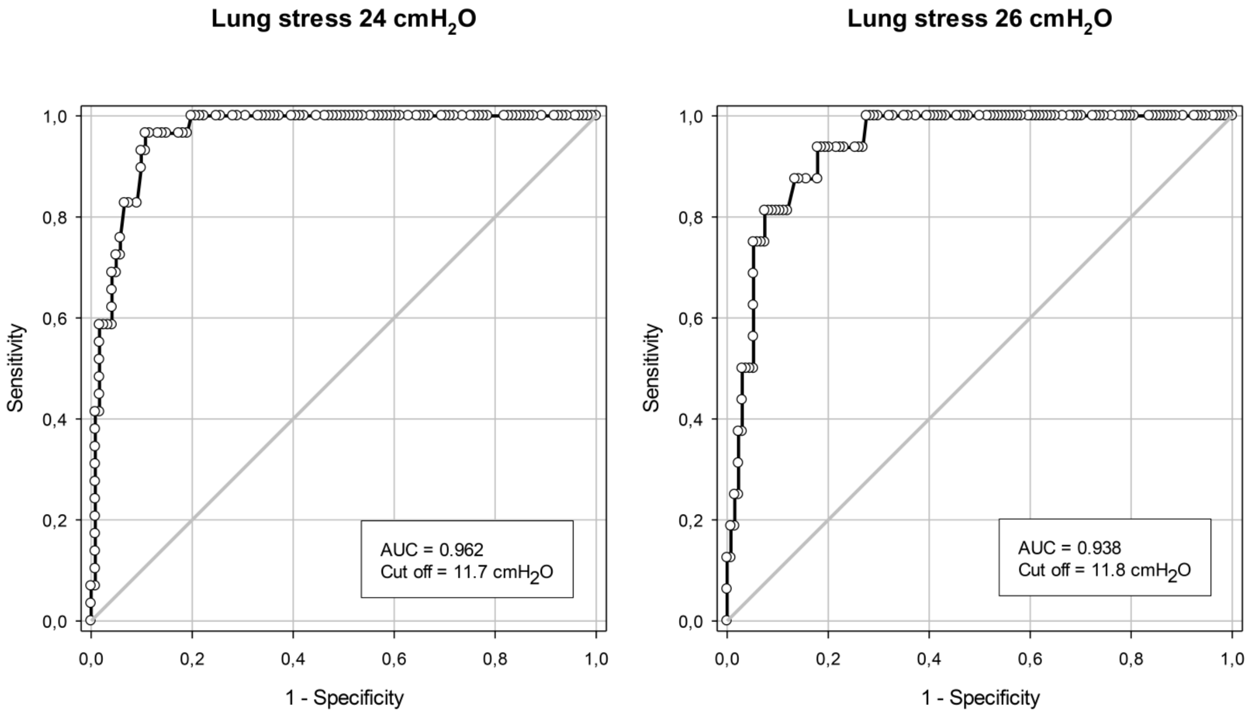

Supplement: Additional file 1: — Expanded results with additional tables and figures. (PDF 2454 kb) [file 13054_2016_1446_MOESM1_ESM.pdf]
